# Supplementary material for: Multi-omics microsampling for the profiling of lifestyle-associated changes in health
Source: Nat Biomed Eng. 2023 Jan 19;8(1):11–29. doi: 10.1038/s41551-022-00999-8 (PMC10805653; doi:10.1038/s41551-022-00999-8)
Supplement: Supplementary file 1 — Supplementary figures, tables and note. [file 41551_2022_999_MOESM1_ESM.pdf]

nature biomedical engineering

Article

https://doi.org/10.1038/s41551-022-00999-8

Multi-omics microsampling for the profiling of lifestyle-associated changes in health

In the format provided by the authors and unedited

Contents

Supplementary Fig. 1 | The comparison between microsampling and intravenous blood sampling approaches.
Supplementary Fig. 2 | Demographics of participants and omics data acquisition for samples.
Supplementary Fig. 3 | Outlier participant detection in the ensure shake study.
Supplementary Fig. 4 | Five metabolic scores.
Supplementary Fig. 5 | Metabolic scores and association between metabolic scores and demographics of participants.
Supplementary Fig. 6 | The wearable and omics data in 24/7 study.
Supplementary Fig. 7 | LOESS regression to smooth multi-omics data in 24/7 study.
Supplementary Fig. 8 | Wearable data can predict the internal molecules (multi-omics data) on an hourly scale.
Supplementary Fig. 9 | Lagged correlation network between wearable and muti-omics data.
Supplementary Fig. 10 | Examples of lagged correlations between CGM and muti-omics data.

Supplementary Note
Content in the supplementary data

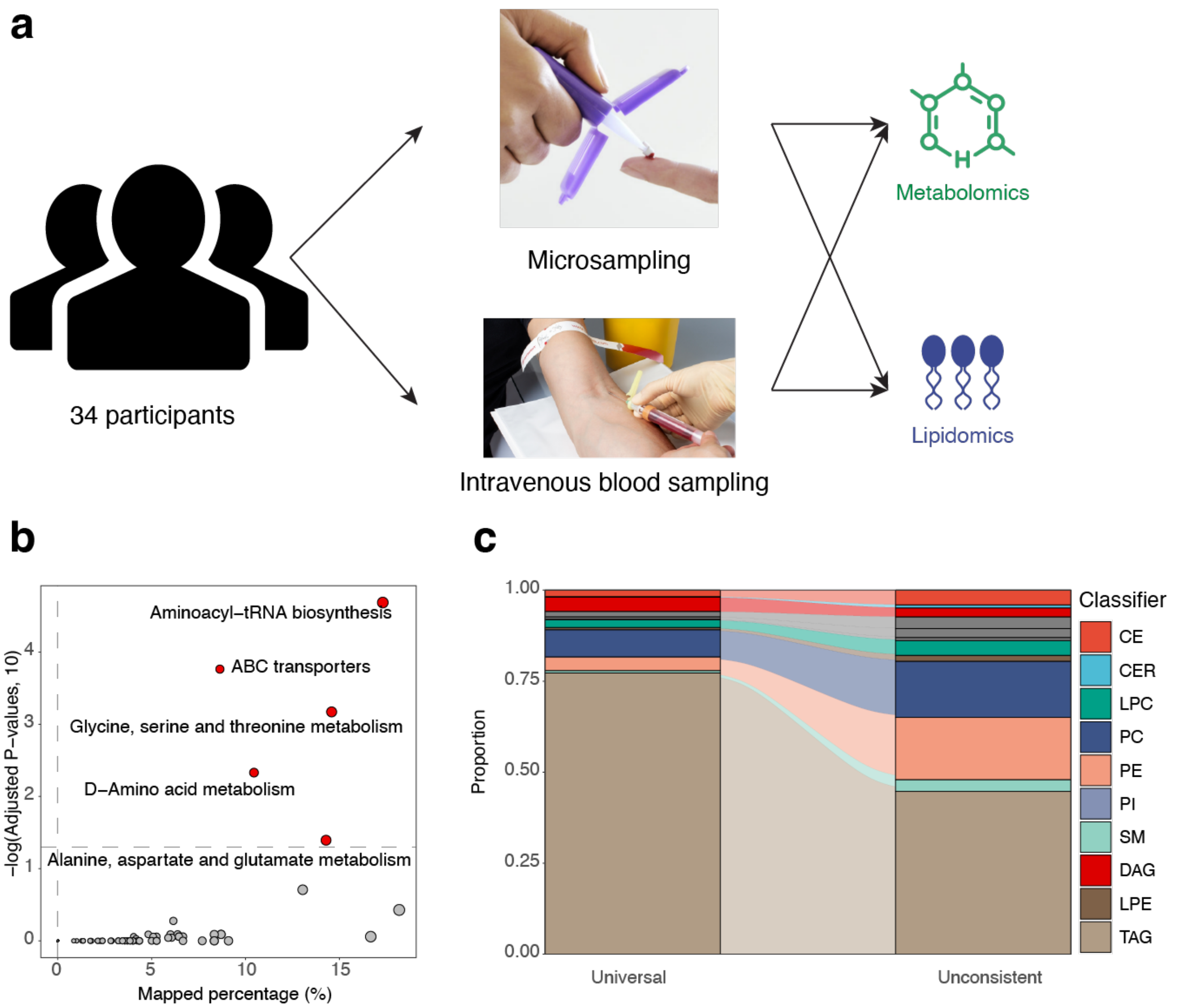

Supplementary Fig. 1 | The comparison between microsampling and intravenous blood sampling approaches. a, 34 participants were recruited and collected for blood microsample and intravenous blood samples, respectively. Then all the samples were used to acquire metabolomics and lipidomics data. **b,** The pathway enrichment result of metabolites that are not correlated well (Spearman correlation < 0.5) between the two approaches. **c,** The enrichment result of lipids that are not correlated well (Spearman correlation < 0.5) between the two approaches. The icons used in this figure are from iconfont.cn/.

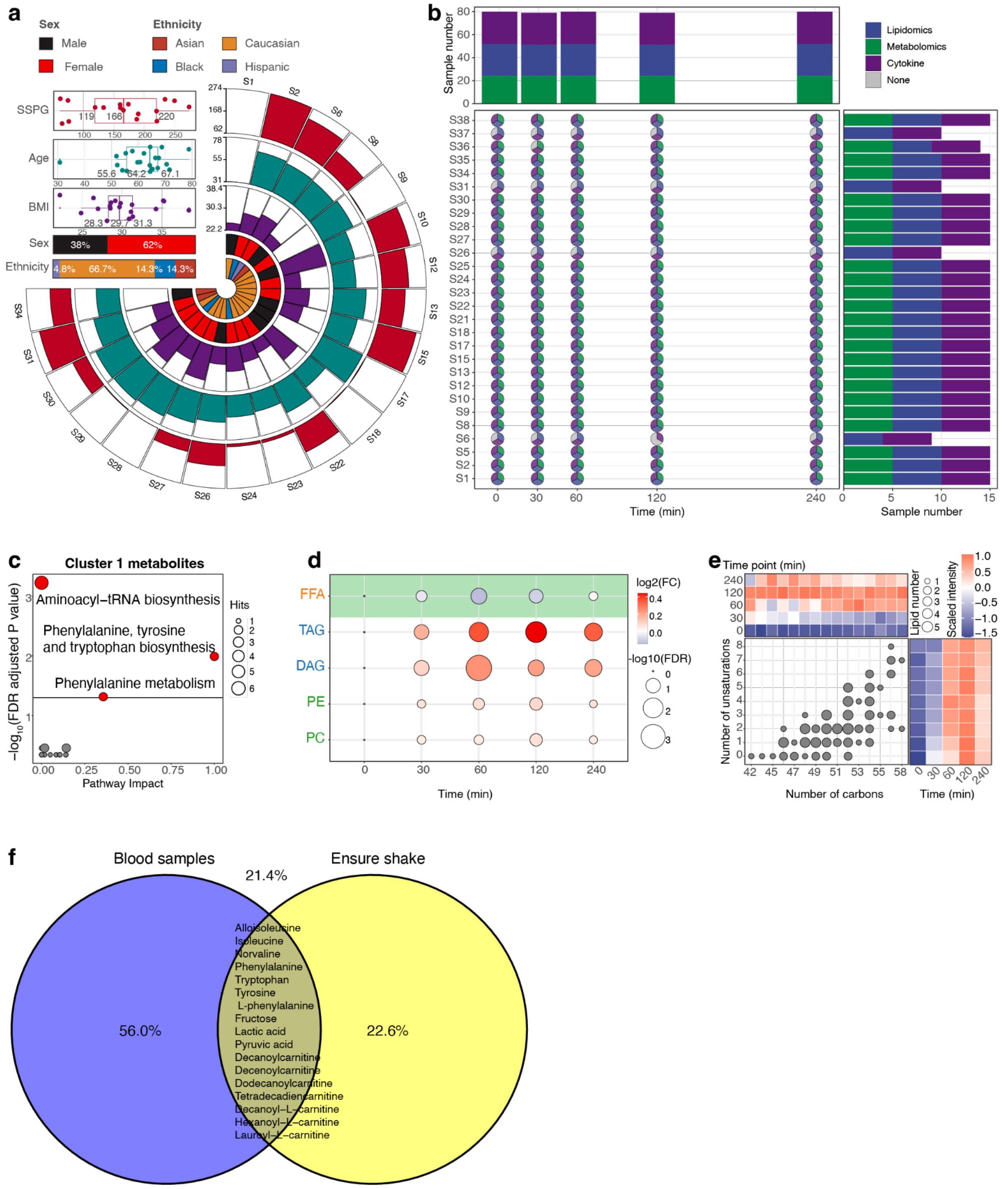

Supplementary Fig. 2 | Demographics of participants and omics data acquisition for samples. a, Demographics of 21 out of 28 participants. SSPG (steady-state plasma glucose), age, BMI (body mass index), sex, and ethnicity are shown using a circular plot. **b**, Omics data acquisition for each participant. Each row is a participant, and each column is a time point. The colors of the pie chart mean the omics data collection. Blue represents the lipidomics data, green represents the metabolomics data, and purple represents the cytokine data. Grey means the corresponding omics data is not acquired **c**, Pathway enrichment result of metabolites in cluster 1. **d**, Different classes of lipids responding to the ensure shake consumption. **e**, TAGs change after consuming ensure shake. **g**, The overlap of compounds from Ensure shake and participants' microsamples.

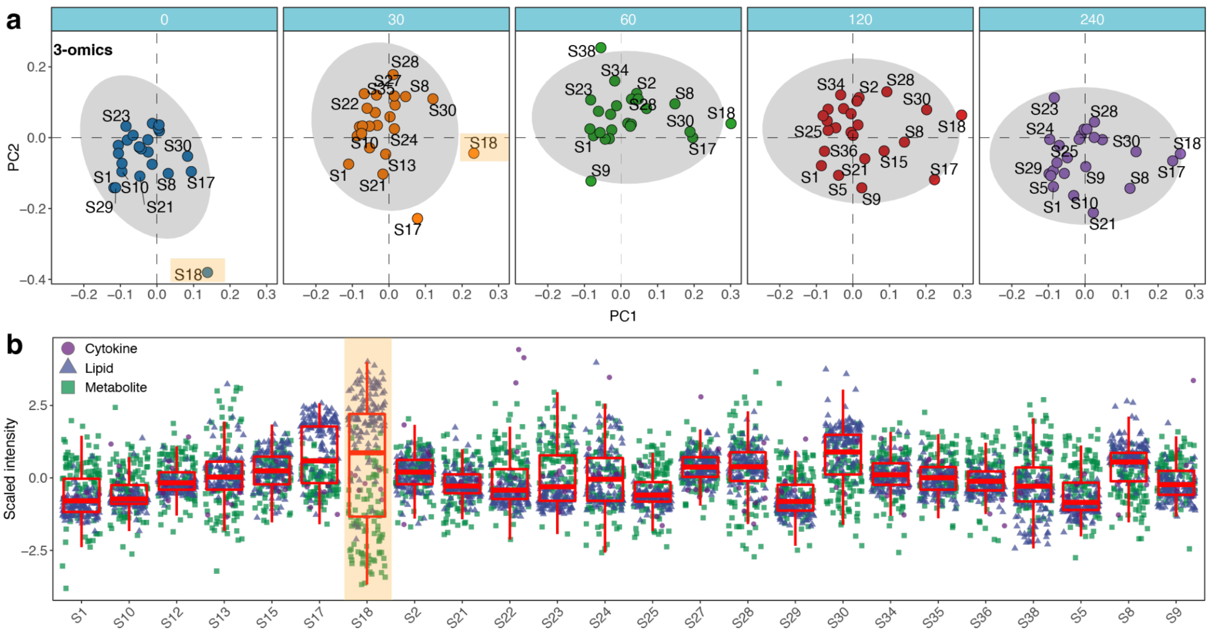

Supplementary Fig. 3 l Outlier participant detection in the ensure shake study. a, PCA score plots to show outlier participants for each time point. The ellipse represents the confidence interval of 95%. **b**, Molecular intensity (scaled) distribution for all the participants in time point 0 (baseline). Colors represent the class of molecules. The bottom whisker represents the minimum, the top whisker represents the maximum, and the square in the box represents the 25%, 50%, and 75% quantile.

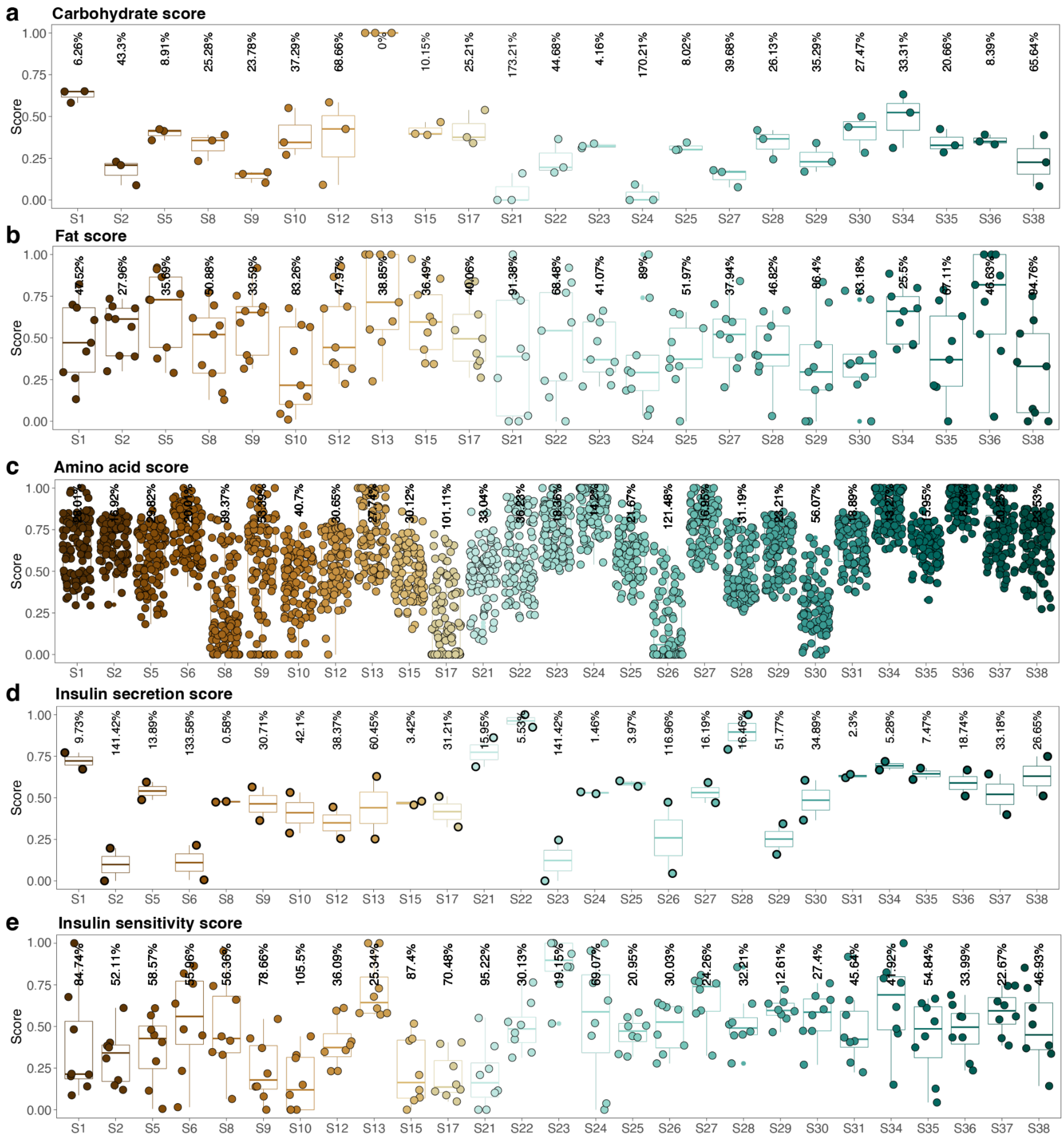

Supplementary Fig. 4 | Five metabolic scores. The normalized scores for each carbohydrate (**a**), fat (**b**), amino acid (**c**), insulin secretion (**d**), and insulin sensitivity (**e**) in each participant. The relative standard deviation (RSD) for each participant is labeled. The bottom whisker represents the minimum, the top whisker represents the maximum, and the square in the box represents the 25%, 50%, and 75% quantile.

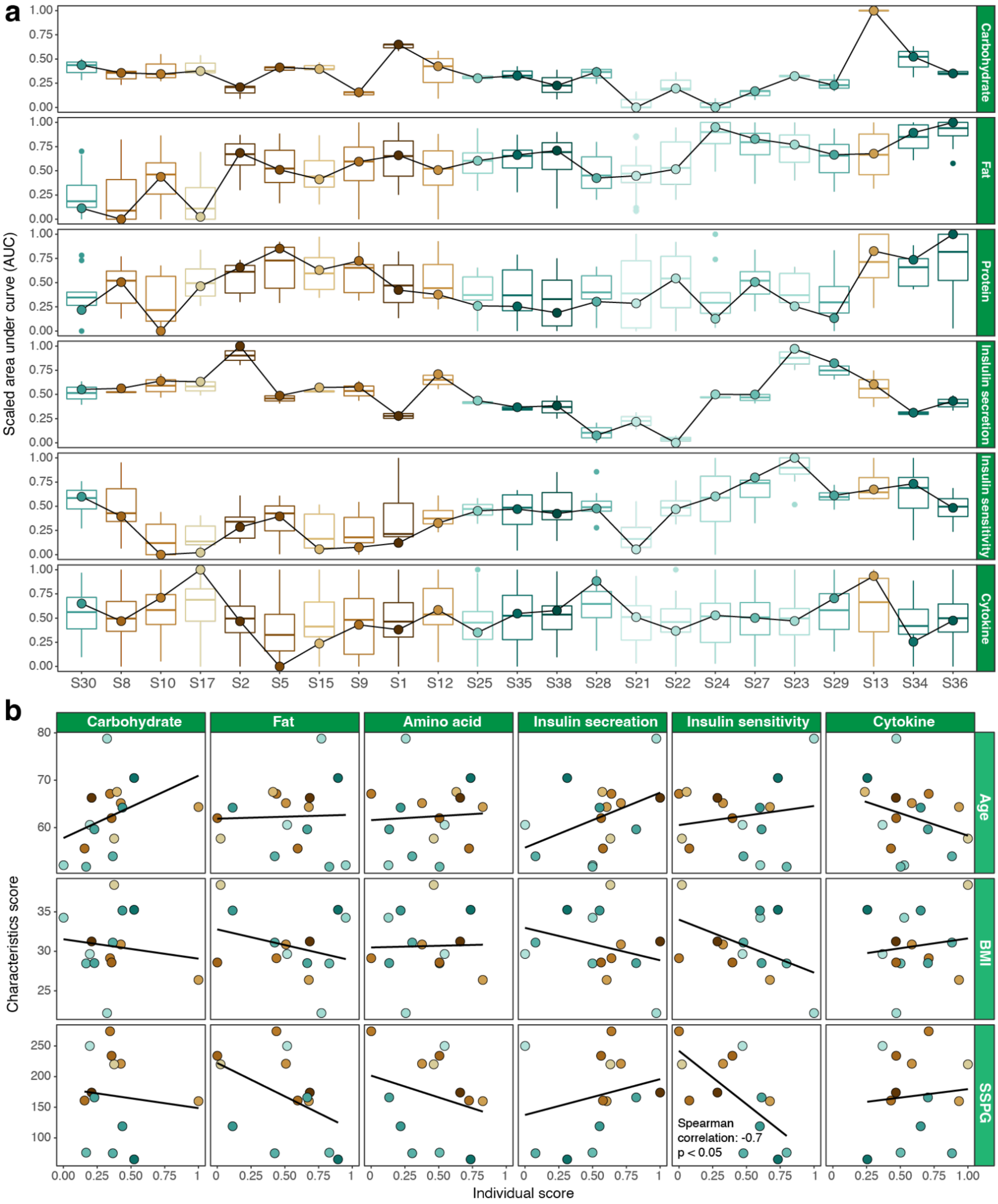

Supplementary Fig. 5 | Metabolic scores and association between metabolic scores and demographics of participants. a, The boxplot to show the metabolic scores for each participant. **b,** The correlation (Spearman) between metabolic scores and age, BMI, and SSPG. The correlation between insulin sensitivity (free fatty acid) is significant, with a correlation of -0.7 and a *p*-value < 0.001. Some studies have demonstrated that elevated plasma levels of free fatty acids are associated with and cause insulin resistance (ref, Elevated free fatty acid level is associated with insulin-resistant state in nondiabetic Chinese people). SSPG (steady-state plasma glucose) is inversely related to insulin sensitivity (SSPG is higher in insulin-resistant subjects and lower in insulin-sensitive subjects). So the negative correlation between SSPG and insulin sensitivity score (calculated using the free fatty acid) is expected. BMI: body mass index. The bottom whisker represents the minimum, the top whisker represents the maximum, and the square in the box represents the 25%, 50%, and 75% quantile.

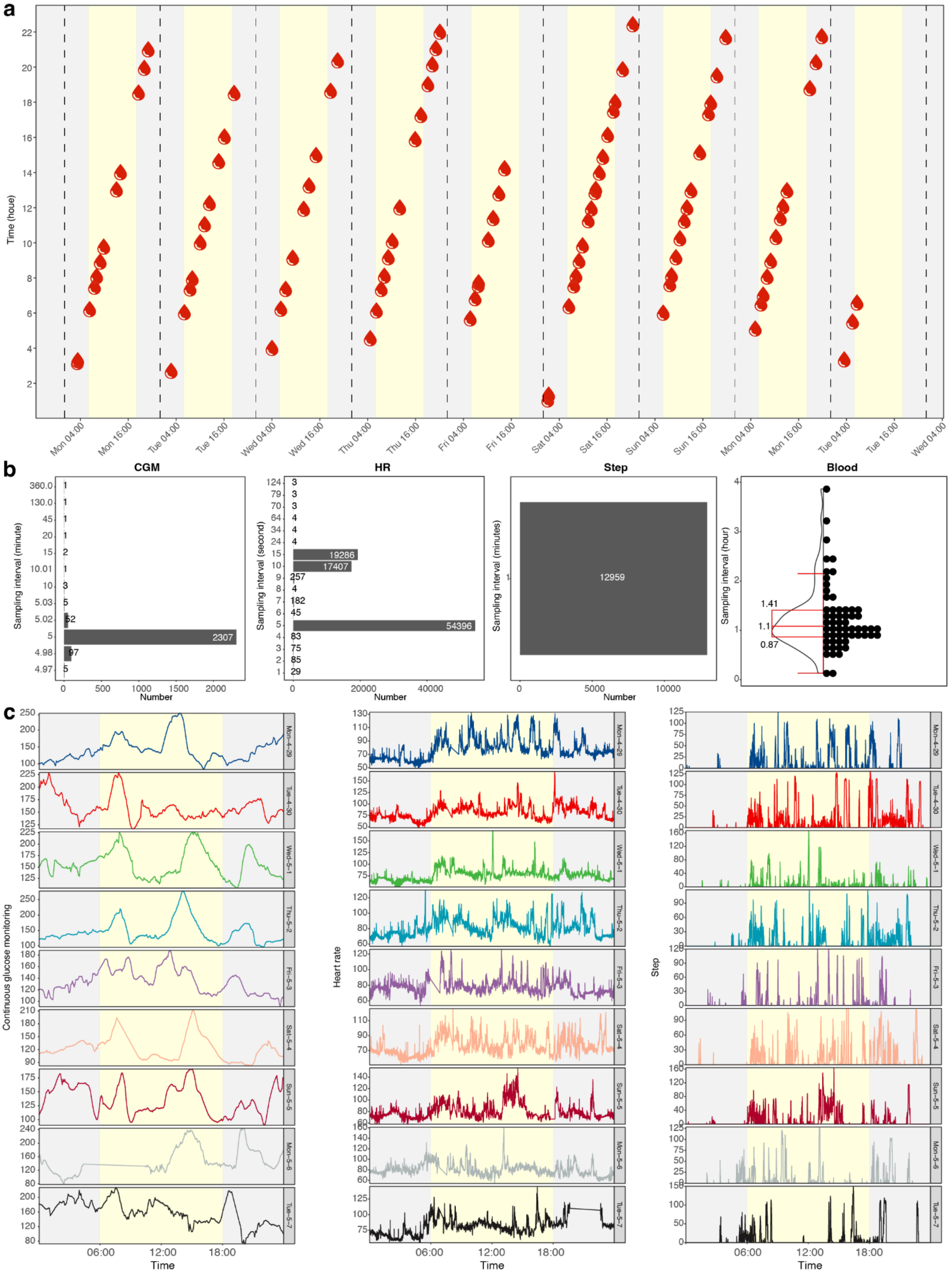

Supplementary Fig. 6 | The wearable and omics data in 24/7 study. a, The distribution of blood microsamples for the participant. **b,** The sampling frequency for continuous glucose monitor glucose (CGM), heart rate (HR), step count, and blood microsamples. **c,** The plots to show wearable data (CGM, heart rate, and step count) each day. The icons used in this figure are from iconfont.cn/.

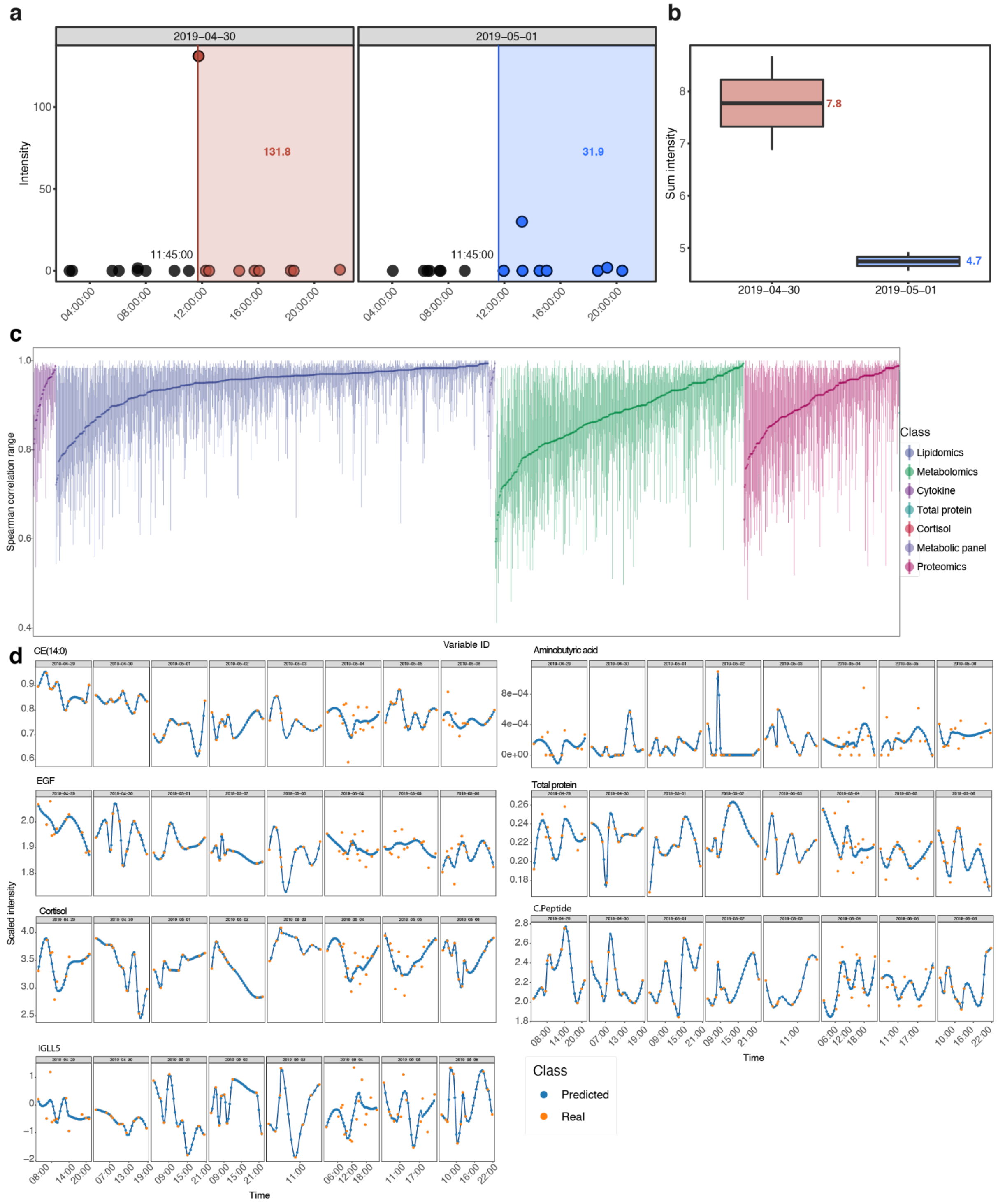

Supplementary Fig. 7 l LOESS regression to smooth multi-omics data in 24/7 study. a, The food logging data (Carbohydrates) of the participant. **b**, The Carbohydrate metabolites (Fructose, Pyruvic acid) intensity distribution on two days. The bottom whisker represents the minimum, the top whisker represents the maximum, and the square in the box represents the 25%, 50%, and 75% quantile. **c,** The Spearman correlations between real intensity and predicted intensity for all the molecules. Most of the correlations > 0.8. The dot color represents the molecular class. **d,** Seven examples for LOESS smoothing for each day. Blue is the predicted data, and orange is the real data.

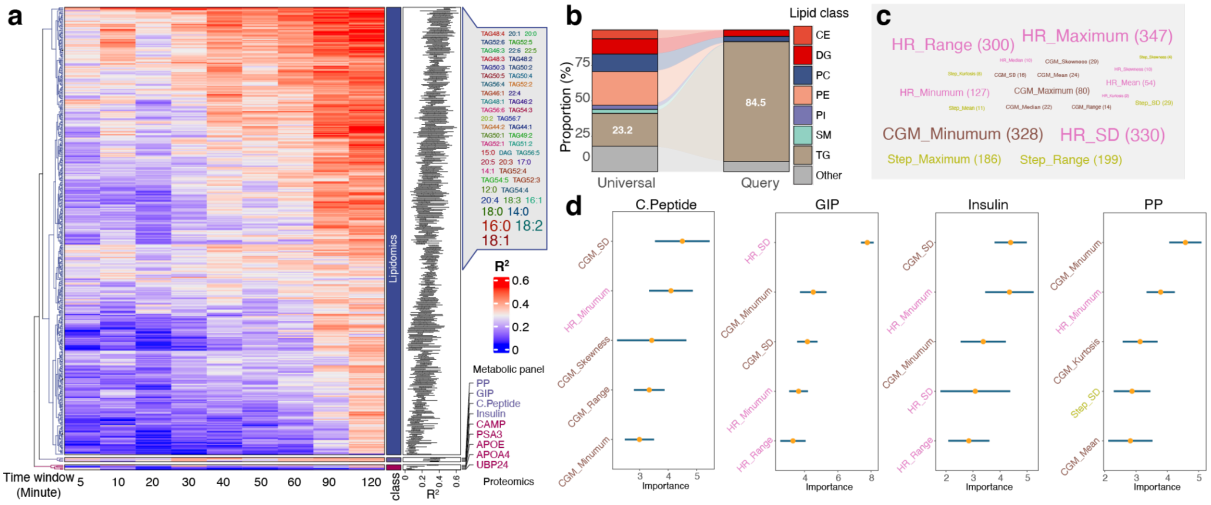

Supplementary Fig. 8 | Wearable data can predict the internal molecules (multi-omics data) on an hourly scale. **a**, Heatmap to show the wearable data to predict internal molecules in different time windows. **b**, Lipid enrichment result. **c**, Wordcloud to show the importance of wearable features in the prediction model to predict lipids. **d**, The importance of wearable features in the prediction model to predict C peptide, GIP, insulin, and PP, respectively.

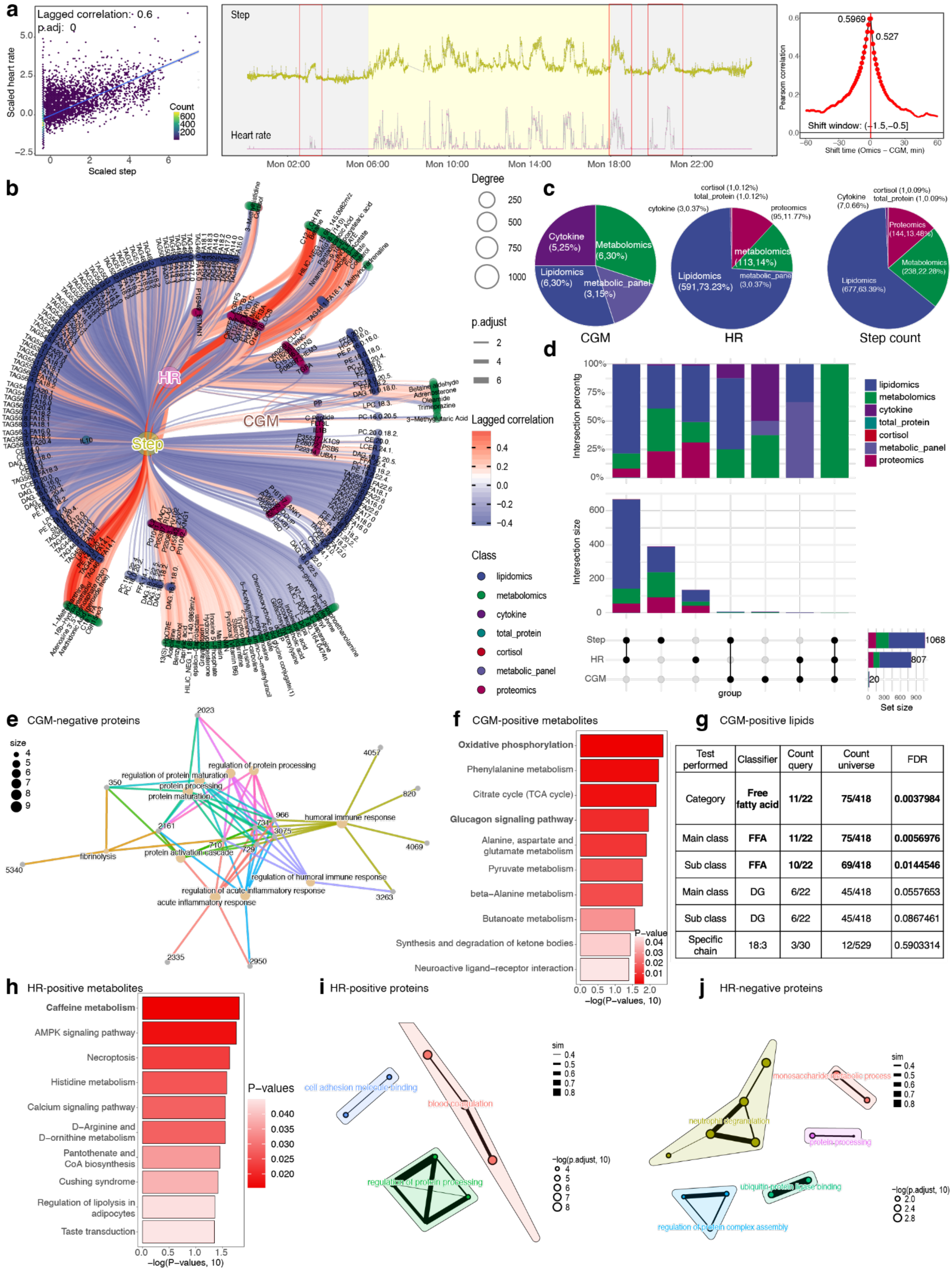

Test performed	Classifier	Count query	Count universe	FDR
Category	Free fatty acid	11/22	75/418	0.0037984
Main class	FFA	11/22	75/418	0.0056976
Sub class	FFA	10/22	69/418	0.0144546
Main class	DG	6/22	45/418	0.0557653
Sub class	DG	6/22	45/418	0.0867461
Specific chain	18:3	3/30	12/529	0.5903314

Supplementary Fig. 9 | Lagged correlation network between wearable and muti-omics data. a, Lagged correlation between step and HR (heart rate). **b**, The whole causal association network between wearable and internal molecules (multi-omics). **c**, The class of molecules connected to the step, HR and CGM, respectively. **d**, The overlap between molecules connected to CGM, heart rate, and step count. **e**, Pathway enrichment results for proteins negatively correlated with CGM. **f**, Pathway enrichment results for metabolites positively correlated with CGM. **g**, Lipid class enrichment results for lipids that positively correlated with CGM. **h**, Pathway enrichment results for metabolites positively correlated with heart rate (HR). **i**, Pathway

enrichment results for proteins positively correlated with heart rate (HR). **j**, Pathway enrichment results for proteins negatively correlated with heart rate (HR).

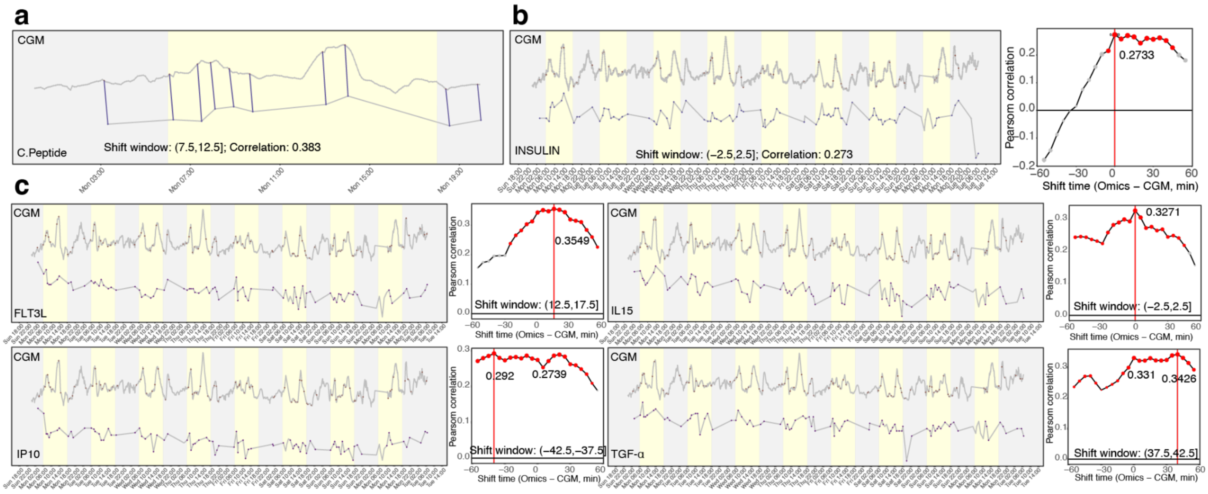

Supplementary Fig. 10 | Examples of lagged correlations between CGM and muti-omics data. a, Laggeted correlation between CGM and C peptide. **b,** Laggeted correlation between CGM and insulin. **c,** Lagged correlation between CGM, FLT3L, IL15, and TGF-α.

Supplementary Note

```
##version
platform       x86_64-apple-darwin17.0
arch           x86_64
os             darwin17.0
system         x86_64, darwin17.0
status
major          4
minor          1.2
year           2021
month          11
day            01
svn rev        81115
language       R
version.string R version 4.1.2 (2021-11-01)
nickname       Bird Hippie

##sessionInfo()
R version 4.1.2 (2021-11-01)
Platform: x86_64-apple-darwin17.0 (64-bit)
Running under: macOS Monterey 12.4

Matrix products: default
LAPACK: /Library/Frameworks/R.framework/Versions/4.1/Resources/lib/libRlapack.dylib

locale:
[1] en_US.UTF-8/en_US.UTF-8/en_US.UTF-8/C/en_US.UTF-8/en_US.UTF-8

attached base packages:
 [1] grid      tcltk     parallel  stats     graphics  grDevices utils     datasets  methods   base

other attached packages:
 [1] wesanderson_0.3.6    ggfortify_0.4.12     Rtsne_0.15           ggiraphExtra_0.3.0
 [5] patchwork_1.1.1      dendextend_1.15.1    GGally_2.1.2         ggforce_0.3.3
 [9] circlize_0.4.14      ComplexHeatmap_2.8.0 corrplot_0.90        Mfuzz_2.52.0
[13] DynDoc_1.70.0        widgetTools_1.70.0   e1071_1.7-8          Biobase_2.52.0
[17] BiocGenerics_0.38.0  forcats_0.5.1        stringr_1.4.0        dplyr_1.0.7
[21] purrr_0.3.4          readr_2.0.0          tidyr_1.1.3          tibble_3.1.3
[25] ggplot2_3.3.5        tidyverse_1.3.1

loaded via a namespace (and not attached):
  [1] utf8_1.2.2             tidyselect_1.1.1       RSQLite_2.2.7          AnnotationDbi_1.54.1
  [5] htmlwidgets_1.5.3      BiocParallel_1.26.1    munsell_0.5.0          codetools_0.2-18
  [9] preprocessCore_1.54.0  withr_2.4.2            colorspace_2.0-2       GOSemSim_2.18.1
 [13] uuid_0.1-4             NLP_0.2-1              ggalluvial_0.12.3      rstudioapi_0.13
 [17] stats4_4.1.2           mzID_1.30.0            labeling_0.4.2         slam_0.1-48
 [21] GenomeInfoDbData_1.2.6 polyclip_1.10-0        bit64_4.0.5            farver_2.1.0
 [25] rprojroot_2.0.2        vctrs_0.3.8            generics_0.1.0         R6_2.5.0
 [29] doParallel_1.0.16      GenomeInfoDb_1.28.1    clue_0.3-59            graphlayouts_0.7.1
 [33] MsCoreUtils_1.9.0      ggiraph_0.7.10         bitops_1.0-7           cachem_1.0.5
 [37] reshape_0.8.8          gridGraphics_0.5-1     assertthat_0.2.1       scales_1.1.1
 [41] vroom_1.5.3            ggraph_2.0.5           gtable_0.3.0           Cairo_1.5-12.2
```

```
 [45] affy_1.70.0            sxtTools_0.99.01        tidygraph_1.2.0         rlang_0.4.11
 [49] systemfonts_1.0.2       mzR_2.26.1              splines_4.1.2           GlobalOptions_0.1.2
 [53] Rdisop_1.52.0           lazyeval_0.2.2          impute_1.66.0           mycor_0.1.1
 [57] broom_0.7.9             reshape2_1.4.4          BiocManager_1.30.16     modelr_0.1.8
 [61] backports_1.2.1         tools_4.1.2             ggplotify_0.0.8         affyio_1.62.0
 [65] ellipsis_0.3.2          RColorBrewer_1.1-2      proxy_0.4-26            MSnbase_2.18.0
 [69] Rcpp_1.0.7              plyr_1.8.6              progress_1.2.2          zlibbioc_1.38.0
 [73] RCurl_1.98-1.3          prettyunits_1.1.1       pbapply_1.4-3           GetoptLong_1.0.5
 [77] viridis_0.6.1           S4Vectors_0.30.0        haven_2.4.1             ggrepel_0.9.1
 [81] cluster_2.1.2           fs_1.5.0                here_1.0.1              magrittr_2.0.1
 [85] masstools_0.99.13       data.table_1.14.0       magick_2.7.2            reprex_2.0.0
 [89] pcaMethods_1.84.0       sjmisc_2.8.7            ggnewscale_0.4.5        ProtGenerics_1.27.2
 [93] matrixStats_0.60.0      hms_1.1.0               XML_3.99-0.6            readxl_1.3.1
 [97] IRanges_2.26.0          gridExtra_2.3           shape_1.4.6             compiler_4.1.2
[101] ncdf4_1.17              crayon_1.4.1            shadowtext_0.0.8        htmltools_0.5.2
[105] proxyC_0.2.0            mgcv_1.8-38             tzdb_0.1.2              RcppParallel_5.1.4
[109] lubridate_1.7.10        DBI_1.1.1               sjlabelled_1.1.8        tweenr_1.0.2
[113] ppcor_1.1               dbplyr_2.1.1            MASS_7.3-54             Matrix_1.3-4
[117] cli_3.0.1               vsn_3.60.0              insight_0.14.2          igraph_1.2.6
[121] pkgconfig_2.0.3         rvcheck_0.1.8           plotly_4.9.4.1          MALDIquant_1.20
[125] xml2_1.3.2              foreach_1.5.1           tkWidgets_1.70.0        simplifyEnrichment_1.5.1
[129] XVector_0.32.0          rvest_1.0.1             digest_0.6.27           Biostrings_2.60.1
[133] tm_0.7-8                cellranger_1.1.0        rjson_0.2.20            nlme_3.1-153
[137] lifecycle_1.0.0         jsonlite_1.7.2          viridisLite_0.4.0       limma_3.48.1
[141] fansi_0.5.0             pillar_1.6.2            ggsci_2.9               lattice_0.20-45
[145] KEGGREST_1.32.0         fastmap_1.1.0           httr_1.4.2              GO.db_3.13.0
[149] glue_1.4.2              png_0.1-7               iterators_1.0.13        bit_4.0.4
[153] class_7.3-19            stringi_1.7.3           blob_1.2.2              org.Hs.eg.db_3.13.0
[157] memoise_2.0.0
```

Content in the supplementary data

Omics data for stability analysis.
Omics data for comparison between microsample and intravenous plasma sample.
Omics data for case study 1.
Altered molecules in case study 1.
Molecules in each cluster in case study 1.
Wearable data for case study 2.
Omics data for case 2.
Circadian molecules in case study 2.
Data for lagged correlation network.
