# Supplementary material for: Multi-omics microsampling for the profiling of lifestyle-associated changes in health
Source: Nat Biomed Eng. 2023 Jan 19;8(1):11–29. doi: 10.1038/s41551-022-00999-8 (PMC10805653; doi:10.1038/s41551-022-00999-8)
Supplement: Supplementary file 2 — Reporting Summary [file 41551_2022_999_MOESM2_ESM.pdf]

nature portfolio

Corresponding author(s): Michael P. Snyder

Last updated by author(s): Dec 2, 2022



# Reporting Summary

Nature Portfolio wishes to improve the reproducibility of the work that we publish. This form provides structure for consistency and transparency in reporting. For further information on Nature Portfolio policies, see our Editorial Policies and the Editorial Policy Checklist.

## Statistics

For all statistical analyses, confirm that the following items are present in the figure legend, table legend, main text, or Methods section.

| n/a | Confirmed | |
|---|---|---|
| ☐ | ☒ | The exact sample size (*n*) for each experimental group/condition, given as a discrete number and unit of measurement |
| ☐ | ☒ | A statement on whether measurements were taken from distinct samples or whether the same sample was measured repeatedly |
| ☐ | ☒ | The statistical test(s) used AND whether they are one- or two-sided<br>*Only common tests should be described solely by name; describe more complex techniques in the Methods section.* |
| ☐ | ☒ | A description of all covariates tested |
| ☐ | ☒ | A description of any assumptions or corrections, such as tests of normality and adjustment for multiple comparisons |
| ☐ | ☒ | A full description of the statistical parameters including central tendency (e.g. means) or other basic estimates (e.g. regression coefficient) AND variation (e.g. standard deviation) or associated estimates of uncertainty (e.g. confidence intervals) |
| ☐ | ☒ | For null hypothesis testing, the test statistic (e.g. *F*, *t*, *r*) with confidence intervals, effect sizes, degrees of freedom and *P* value noted<br>*Give P values as exact values whenever suitable.* |
| ☒ | ☐ | For Bayesian analysis, information on the choice of priors and Markov chain Monte Carlo settings |
| ☒ | ☐ | For hierarchical and complex designs, identification of the appropriate level for tests and full reporting of outcomes |
| ☒ | ☐ | Estimates of effect sizes (e.g. Cohen's *d*, Pearson's *r*), indicating how they were calculated |

*Our web collection on statistics for biologists contains articles on many of the points above.*

## Software and code

Policy information about availability of computer code

| | |
|---|---|
| Data collection | ProteoWizard: Version. 3.0.19314-fb982f15b |
| Data analysis | R: 4.1.2; Rstudio: 2021.09.2. R package: colorspace_2.0-2 rjson_0.2.21 ellipsis_0.3.2 leaflet_2.1.0 rprojroot_2.0.2 circlize_0.4.14 GlobalOptions_0.1.2 clue_0.3-60 rstudioapi_0.13 mzR_2.28.0 affyio_1.64.0 fansi_1.0.2 xml2_1.3.3 codetools_0.2-18 ncdf4_1.19 doParallel_1.0.17 impute_1.68.0 knitr_1.37 jsonlite_1.7.3 cluster_2.1.2 vsn_3.62.0 png_0.1-7 readr_2.1.2 compiler_4.1.2 httr_1.4.2 assertthat_0.2.1 fastmap_1.1.0 lazyeval_0.2.2 limma_3.50.0 cli_3.2.0 htmltools_0.5.2 tools_4.1.2 gtable_0.3.0 glue_1.6.1 affy_1.72.0 dplyr_1.0.8 Biobase_2.54.0 cellranger_1.1.0 jquerylib_0.1.4 iterators_1.0.14 crosstalk_1.2.0 stringr_1.4.0 openxlsx_4.2.5 MSnbase_2.20.4 pcaMethods_1.86.0 hms_1.1.1 ProtGenerics_1.26.0 parallel_4.1.2 RColorBrewer_1.1-2 ComplexHeatmap_2.10.0 yaml_2.3.4 pbapply_1.5-0 yulab.utils_0.0.4 sass_0.4.0 stringi_1.7.6 highr_0.9 S4Vectors_0.32.3 foreach_1.5.2 BiocGenerics_0.40.0 zip_2.2.0 BiocParallel_1.28.3 shape_1.4.6 systemfonts_1.0.3 rlang_1.0.1 pkgconfig_2.0.3 matrixStats_0.61.0 mzID_1.32.0 evaluate_0.15 lattice_0.20-45 purrr_0.3.4 htmlwidgets_1.5.4 tidyselect_1.1.1 here_1.0.1 ggsci_2.9 plyr_1.8.6 bookdown_0.24 R6_2.5.1 IRanges_2.28.0 generics_0.1.2 DBI_1.1.2 pillar_1.7.0 withr_2.4.3 MsCoreUtils_1.6.0 tibble_3.1.6 crayon_1.5.0 utf8_1.2.2 plotly_4.10.0 tzdb_0.2.0 readxl_1.3.1 data.table_1.14.2 webshot_0.5.2 digest_0.6.29 tidyr_1.2.0 gridGraphics_0.5-1 ggplotify_0.1.0 bslib_0.3.1 |

For manuscripts utilizing custom algorithms or software that are central to the research but not yet described in published literature, software must be made available to editors and reviewers. We strongly encourage code deposition in a community repository (e.g. GitHub). See the Nature Portfolio guidelines for submitting code & software for further information.

# Data

Policy information about availability of data

All manuscripts must include a data availability statement. This statement should provide the following information, where applicable:

- Accession codes, unique identifiers, or web links for publicly available datasets
- A description of any restrictions on data availability
- For clinical datasets or third party data, please ensure that the statement adheres to our policy

All the data used in this study are provided as Supplementary Data.

# Human research participants

Policy information about studies involving human research participants and Sex and Gender in Research.

| | |
|---|---|
| Reporting on sex and gender | We have reported the sex information on the enrolled patients, which is determined by self-reporting |
| Population characteristics | Ensure shake study cohort: 21 out of 28 participants have completed demographic data. The median steady-state plasma glucose (SSPG) was 166, the median age was 64.2 years, and the median body mass index (BMI) was 29.7. Among all the participants, 38% were male, and 14.3% were Asian, 14.3% Black, 66.7% Caucasian and 4.8% Hispanic. |
| Recruitment | Ensure shake study cohort: Twenty-eight participants were enrolled in the Ensure shake study under an institutional review board (IRB)-approved protocol (IRB-47966 at Stanford University) with written consent. 21 out of 28 participants have completed demographic data.<br><br>24/7 study cohort: Only one participant (male, 64 years old) was enrolled in the 24/7 study under an IRB-approved protocol (IRB-23602 at Stanford University) with written consent. The participant was instructed to perform self-collected finger prick microsamples approximately every hour during waking hours and overnight periods sporadically for 7 days. |
| Ethics oversight | The study protocol was approved by the institutional review board at Stanford University (protocols IRB-47966 and IRB-23602). |

Note that full information on the approval of the study protocol must also be provided in the manuscript.

# Field-specific reporting

Please select the one below that is the best fit for your research. If you are not sure, read the appropriate sections before making your selection.

☒ Life sciences ☐ Behavioural & social sciences ☐ Ecological, evolutionary & environmental sciences

For a reference copy of the document with all sections, see nature.com/documents/nr-reporting-summary-flat.pdf

# Life sciences study design

All studies must disclose on these points even when the disclosure is negative.

| | |
|---|---|
| Sample size | No statistical methods were used to calculate the sample size. For the shake study, 28 participants were enrolled, and for the 24/7 study, 1 participant was enrolled. |
| Data exclusions | No data were excluded. |
| Replication | No replication was carried out. |
| Randomization | For all datasets, samples were assigned randomly to acquire omics data. |
| Blinding | The investigators were blinded to group information during data collection. At the time of sample acquisition and processing, the scientists were completely unaware of the sample group. |

# Reporting for specific materials, systems and methods

We require information from authors about some types of materials, experimental systems and methods used in many studies. Here, indicate whether each material, system or method listed is relevant to your study. If you are not sure if a list item applies to your research, read the appropriate section before selecting a response.

## Materials & experimental systems

| n/a | Involved in the study |
|---|---|
| ☒ | ☐ Antibodies |
| ☒ | ☐ Eukaryotic cell lines |
| ☒ | ☐ Palaeontology and archaeology |
| ☒ | ☐ Animals and other organisms |
| ☒ | ☐ Clinical data |
| ☒ | ☐ Dual use research of concern |

## Methods

| n/a | Involved in the study |
|---|---|
| ☒ | ☐ ChIP-seq |
| ☒ | ☐ Flow cytometry |
| ☒ | ☐ MRI-based neuroimaging |
